# Computer-Assisted Porcelain Laminate Veneer Preparation: A Scoping Review of Stereolithographic Template Design and Fabrication Workflows

**DOI:** 10.3390/dj12100302

**Published:** 2024-09-25

**Authors:** Xin Guan, Yew Hin Beh, In Meei Tew

**Affiliations:** Department of Restorative Dentistry, The Faculty of Dentistry, The National University of Malaysia, Bangi 50300, Malaysia; p138226@siswa.ukm.edu.my (X.G.); behyewhin@ukm.edu.my (Y.H.B.)

**Keywords:** dental esthetic, tooth reduction, CAD/CAM, veneers

## Abstract

Computer-assisted preparation of porcelain laminate veneers (PLVs) using stereolithographic templates has been developed to enhance the accuracy of tooth preparation. However, the digital workflows involved in guided PLV preparation remain inconsistently defined across various practices. Therefore, this scoping review aimed to examine publications on computer-assisted PLV preparation to identify the key stage of digital workflows involved in designing and fabricating stereolithographic templates, as well as to highlight the limitations of various template designs. This scoping review aimed to identify publications on digital workflows for designing and fabricating stereolithographic templates in computer-assisted porcelain laminate veneer preparation. A systematic search on MEDLINE/PubMed, Web of Science and Scopus identified English-language articles published from 2014 to March 2024. Eligible articles focused on digitally designed and fabricated tooth reduction templates for porcelain laminate veneers, excluding conventional tooth preparation procedures for tooth reduction assessment. Seven clinical reports were included, demonstrating various 3D data acquisition techniques for virtual patient generation. All articles described virtual diagnostic wax-ups on digital casts, with two using a virtual articulator. Only five articles documented chair-side mock-ups with resin trial restorations to evaluate planned dental esthetics. Additionally, virtual tooth preparation prior to templates design was included in only four articles. The templates were designed using different software and ranged from simple designs with access windows to complex stacked templates with rotary instrument sleeved windows. Each template design had limitations affecting tooth reduction accuracy. All articles reported printing templates in clear acrylic resin using different technologies. In conclusion, the review highlights a lack of standardization in the digital workflow for designing stereolithographic templates for PLVs. Establishing a sound protocol for designing the tooth reduction templates is essential to ensure the accuracy and consistency of veneer preparation.

## 1. Introduction

The porcelain laminate veneer (PLV) is a minimally invasive approach for the esthetic rehabilitation of anterior teeth [1]. The long-term success of PLVs depends on a conservative tooth preparation within enamel for achieving an optimal bonding strength with ceramic veneers [2]. Therefore, it is essential to precisely control the tooth preparation depth to ensure enamel preservation [3].

A conventional workflow for controlling tooth reduction depths in veneer preparation incorporates several techniques, including using depth grooves, silicone indices or a thermoplastic guide [4]. However, these techniques can have limitations, such as variability in depth control, potential inaccuracies and over-reliance on the skill and experience of the dentist which may compromise the longevity of PLVs [5]. With advancements in digital technology, computer-aided design and computer-aided manufacturing (CAD-CAM) have made computer-assisted tooth preparation a reality [6]. This digital workflow incorporates a virtual tooth preparation planning into the design of stereolithographic templates, which are then printed and used for clinical execution, ensuring higher accuracy and consistency in veneer preparation.

The workflow in designing and manufacturing stereolithographic templates for PLV preparation involves several main stages. In the initial stage, digital diagnostic data are obtained either directly through intraoral scans, or indirectly via laboratory scans of diagnostic casts [7]. These three-dimensional data are used to generate a virtual patient model for further assessment and veneer preparation planning. In the second stage, a virtual diagnostic wax-up is performed to simulate the desired esthetic outcome. This virtual wax-up is essential for fabricating provisional restorations, allowing for chairside esthetic validation and adjustments [8]. In the third stage, virtual tooth preparation is performed according to the planned reduction depth. The virtual plan is then used for the design of the tooth reduction templates, which are printed out for precise tooth preparation for PLV for the final step [9]. 

The digital workflow of computer-assisted tooth reduction for PLVs has been well reported; however, there is variability in the designs of stereolithography templates produced by different CAD software [6,10,11], which can impact the precision and esthetic outcomes of tooth preparation for PLVs. Moreover, more recent publications have included the use of face scanners and extraoral and intraoral photographs in the digital workflow to provide more comprehensive virtual diagnostic data for smile design [12,13,14]. Therefore, this scoping review aimed to map the existing evidence, and provide a more comprehensive understanding of the current state of technology and practices in computer-assisted tooth reduction for PLVs. This will ultimately contribute to the development of standardized protocols and best practices, ensuring optimal patient outcomes in esthetic dentistry.

## 2. Materials and Methods

This scoping review was conducted in accordance with the guidelines of the Preferred Reporting Items for Systematic Reviews and Meta-analysis Extension for Scoping Reviews (PRISMA-ScR) checklist [15,16,17]. The review protocol was registered and can be accessed at http://doi.org/10.17605/OSF.IO/CP3S2. In this scoping review, two research questions were addressed:

- What are the key steps involved in the digital workflow for designing and manufacturing stereolithographic templates in computer-assisted PLV preparation?
- What are the limitations of stereolithographic template designs that may impact the tooth preparation accuracy?

### 2.1. Literature Search

A search strategy was conducted through PubMed, Web of Science and Scopus databases from 2014 to Mac 2024 using keywords as listed in Table 1.

The inclusion criteria were as follows:

(i) Articles detailing a digital workflow for designing and fabricating stereolithographic templates in computer-assisted PLV preparation.
(ii) Clinical studies involving the preparation of PLV for six or more teeth.
(iii) Articles published in English.

Studies were excluded based on the following criteria:

(i) Articles focusing on conventional veneer preparation procedures.
(ii) Articles using templates for tooth reduction assessment.
(iii) In vitro studies and review articles.

### 2.2. Data Extraction

The titles and abstracts were initially screened by two independent reviewers (G.X and T.I.M) to ensure that the studies satisfied the inclusion criteria, and a matrix of relevant data was produced. Any disagreement about inclusion was resolved by discussion. The full text of included studies was read independently by the same two independent reviewers. After identifying articles that met the inclusion criteria, the following data were extracted and tabulated: main author’s name, publication year, study design, number of teeth involved for veneer preparation, types of scanners, STL diagnostic data acquisition, virtual patient generation method, virtual diagnostic wax-up, chairside esthetic validation procedure, CAD software for designing stereolithographic template, virtual tooth preparation, number of stereolithographic templates, designs of stereolithographic template, types of three-dimensional (3D) printer and resins, and limitations of the template design.

### 2.3. Quality Assessment

The Joanna Briggs Institute (JBI) critical appraisal checklist for case reports and JBI criterial appraisal checklist for case series [18] was used for quality assessment of the case reports and case series respectively. Two reviewers (G.X and T.I.M) performed the quality assessment independently. Disagreements were resolved through discussion to ensure consensus. The evaluation process focused on eight different elements for case reports and ten different elements for case series, as outlined in the respective JBI checklists. Each element was assessed and scored as “Yes”, “No”, “Unclear” or “Not Applicable” depending on the availability and clarity of the information provided in the study. 

## 3. Results

**Figure 1:**
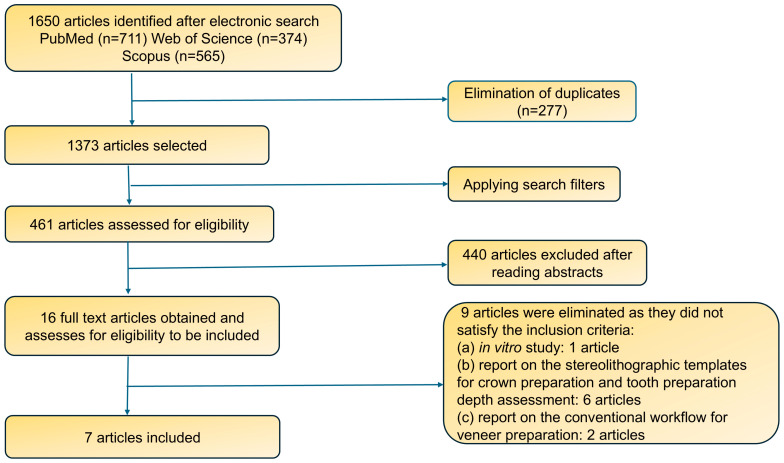
Flow chart of screening and selection process.

From the initial database search, 1650 articles were identified. Duplicate entries were removed using a reference manager (Endnote X9; Clarivate, San Diego, CA, USA), resulting in 1373 articles. After applying search filters, 912 papers were excluded, leaving 461 articles. Based on the title and abstract, 440 articles were excluded, with 16 articles retrieved for eligibility assessment. The full-text versions of these 16 articles were scrutinized for their content. Nine articles were eliminated for various reasons: they were not clinical studies [19], reported on stereolithographic templates for crown preparation [20,21,22,23], did not provide information on the digital design and fabrication of stereolithographic templates for veneer preparation [24,25] and focused on the use of stereolithographic templates to assess tooth preparation depth [26,27]. Only seven articles met the criteria and were analyzed. A flow chart of the screening and selection process is shown in Figure 1.

The characteristics of the included case reports and workflow of the virtual patient model and the esthetic pre-evaluative temporary approach in computer-assisted PLV preparation are shown in Table 2. All studies recruited patients who were candidates for esthetic rehabilitation involving six to sixteen teeth using PLV. The technique for acquiring clinical three-dimensional data varied among the included articles. One article [10] favored intraoral scanning, another two article [28,29] reported indirect scanning using laboratory scanner, while one case series employed both intraoral and extraoral scanning for two distinct cases. The remaining three [11,30,31] articles utilized a combination of face scanners, cone-beam computed tomography scans and intraoral scanners. For generating a virtual patient, three articles [6,28,29] reporting the use of maxillary scans, while the remaining four article [10,11,30,31] superimposed maxillary scans with photographs or face scans alongside cone-beam computed tomography scans. Regarding virtual diagnostic wax-up, five articles [6,10,11,28,29] described performing the procedure on digital casts following esthetic parameters, while the other two articles [30,31] reported using a virtual articulator additionally to verify the functionality of diagnostic wax-up. Only five articles documented chair-side mock-ups based on printed diagnostic wax casts using resin-type trial restoration to validate the planned esthetics. 

The workflow of designing and manufacturing tooth reduction stereolithographic templates, along with their limitations are detailed in Table 3. The software used for designing stereolithographic templates varied among the included articles. Four articles [28,29,30,31] favored Exocad dental software, while other three used Meshmixer [10], First fit [6] and 3Shape [11] software (Exocad 2.4 and Exocad 2018) respectively. Prior to designing templates, only four articles [6,10,11,31] incorporated virtual tooth preparation into the digital workflow. Most articles reported fabricating a single template while the other two articles [6,10] reported designing a series of templates for step-by-step tooth preparation. All articles reported printing the templates using resin with various types of 3D printers. Each template design presented with limitations; for example, cross-shaped designs were discussed in two articles [28,29], while other articles [30,31] discussed the requirement of using a calibrated bur with a stopper to create depth grooves. The first-fit system technique, applied in one article [6], lacked guidance for tooth reduction in interproximal areas and the cervical finish line and required a specially designed bur. Additionally, templates with an open window necessitate free-hand preparation at the cervical finish line [11]. A series of templates with a rotary instrument access sleeve design [10] required free-hand polishing after guided tooth preparation.

The results of the JBI critical appraisal checklist for the case reports and the JBI critical appraisal checklist for the case series are summarized in Table 4; Table 5. Overall, the study quality of the included studies was moderate-to-good, with most of the case reports scoring between 6 and 8.

## 4. Discussion

**Figure 2:**
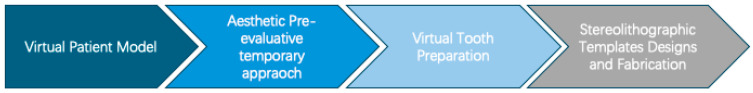
Four key stages in digital workflow of designing and fabricating stereolithographic templates in computer-assisted veneer preparation.

The importance of computer-assisted PLV preparation in controlling the depth of enamel reduction has been well established by previous studies [3,32]. However, there is variation in the use of digital technologies and stereolithographic templates designs within the digital workflow of veneer preparation. Therefore, this scoping review outlined the four key stages involved in computer-assisted PLV preparation for at least six anterior teeth for comprehensive esthetic rehabilitation (Figure 2) and highlighted the limitations associated with different stereolithography template designs which impact the accuracy of tooth preparation. 

### 4.1. Three-Dimensional Diagnostic Data Acquisition and Virtual Patient Model Generation

Generating a virtual patient model (VPM) is a crucial initial step in computer-assisted PLV preparation. Various techniques have been developed for this purpose. Tinoco et al. and Robles et al. reported a simple technique to create VPM from direct or indirect scans of maxillary and mandibular dentition, but this VPM offers limited details for planning the guided veneer preparation [28,29]. In contrast, comprehensive VPM, integrating intraoral scans or CBCT scans with facial scans with a full smile have been well documented following the recent emergence of facial scanners [30,31]. The intraoral scans, facial scans and CBCT scans are exported into the dental computer-assisted design (CAD) software. All the data are then merged using teeth as reference landmarks to generate a VPM [32]. This 3D representation of the actual patient provides additional esthetic details, such as facial profile, lip–teeth relationship, smile line and lip mobility. These additional details are essential not only for precise esthetic planning but also for designing and fabricating tooth reduction templates [33]. Alternatively, advancements in computer-assisted design (CAD) software now enable for a cost-effective approach to generating comprehensive VPMs by superimposing two-dimensional extraoral photographs on three-dimensional intraoral scans using the remaining teeth as merging landmarks [10,11]. However, achieving optimal image-to-geometry mapping registration requires adherence to specific criteria during photograph acquisition such as a frontal view of patients for full-face images with lips relaxed and smiling, lower one-third face images, and an optional image of full face with retractors for enhanced visibility [34].

### 4.2. Virtual Diagnostic Wax-Up and Esthetic Pre-Evaluative Temporary Approach

Esthetic planning is a crucial subsequent stage in PLV preparation, facilitated by a diagnostic wax-up. The advancement of CAD-CAM technology has greatly enhanced the treatment planning efficacy and the predictability of desired outcomes by enabling digital performance of the diagnostic wax-up on VPMs. The CAD software solution commonly used in the included studies such as Exocad [10,29,30,31], 3shape Trio’s software [11] and first fit software [6] are equipped with digital smile design (DSD) features for analyzing facial, dentogingival and dental esthetics proportions. The key esthetic and functional parameters considered in dental esthetic planning include facial midline, smile cant, lower lip line, buccal corridor, gingival line and other additional parameters [35,36]. These parameters are critical for achieving personalized and esthetically pleasing results in definitive PLVs. The virtual esthetic smile can be digitally manipulated using the templates from a library of pre-designed shapes and sizes in the CAD software. To further enhance outcome predictability, Luo et al. and Gao et al. emphasize the importance of occlusal analysis and report on integrating the virtual articulator into the VPM for smile design [30,31].

An esthetic pre-evaluation through a chairside mock-up is crucial for assessing the planned smile design, phonetics and function of planned PLVs as well as facilitating communication between clinicians and patients. Typically, the mock-up involves using bis-acylic based trial restorations fabricated from the silicone index based on printed wax casts. However, Figueira et al. proposed an alternative method known as Bonded Functional Esthetic Prototype (BFEP) [11]. In this approach, bis-acryl trial restorations were fabricated according to the digital wax-up, but the trial restorations were bonded to the tooth surface using a spot-etched technique for clinical try-in. Subsequent tooth preparation was performed through the BFEP to achieve a predictable treatment outcome aligned with the digital plan. This BFEP approach differs from the technique suggested by McLean, in which highly filled, flowable composite resins are bonded to minimally prepared enamel with the aid of a clear template [34]. This technique allows the mock-up to potentially serve as long-term provisional restorations, enabling evaluation of patient’s response to the proposed treatment before proceeding with the definitive treatment. Upon completing the mock-up, a final intraoral scan is required to proceed with designing stereolithographic templates, especially if adjustments are required to the trial restorations.

### 4.3. Virtual Tooth Preparation

Virtual preparation is an optional step within a digital workflow for PLV preparation. The virtual tooth preparation procedure as suggested by a several studies allows for precise calculation of the required tooth preparation depth from the digital wax-up. The calculated depth is then integrated into the stereolithographic template design to guide the tooth reduction accurately. Virtual preparation can be accomplished by using the “off-set” tool in the CAD software to reduce the desired volume over the digital wax-up, considering the minimal required veneer thickness at the incisal edge, middle and cervical regions of tooth’s labial surfaces, as well as finishing line [10,31]. On the other hand, a more precise virtual tooth preparation that simulates chair-side tooth preparation using virtual burs has been reported by Figueira et al. [11]. This virtual preparation enables the creation of an ideal space for chair-side tooth preparation, ensuring uniform restorative spaces to achieve the desired final color in the PLV. Additionally, the precise virtual tooth preparation enabled by the First-fit system facilitates a one-stage approach, in which veneers are designed and fabricated following the virtual tooth preparation, allowing them to be ready for immediate cementation once the actual tooth preparation is completed [6].

### 4.4. Stereolithographic Template Designs

Different designs of the tooth reduction stereolithographic templates have been suggested in the included studies to guide PLV preparation; each has limitations that affect the accuracy of tooth reduction to varying degrees. Figueira et al. described a simple template featuring an open-window design that does not cover the labial surface and incisal edge of the tooth. This design allows for the refinement of the incisal and labial reduction after initial hands-free veneer preparation. While this window guide facilitates some aspects of the tooth reduction procedure, it provides limited control over bur movement, which may increase the risk of over tooth preparation [11]. 

Tinoco et al. and Robles et al. developed a simple cross-shaped template, designed to assist in establishing initial vertical and horizontal depth grooves using a round diamond bur. The vertical groove extends from the midpoint of the incisal edge to the midpoint of the cervical finish line, while the horizontal depth groove runs from the mesial to distal finish line at the middle of the labial surfaces. The prepared depth grooves were used to guide the subsequence free-hand tooth preparation after removal of the template. Although this type of template provides limited comprehensive guidance for tooth preparation [28,29], it serves as a useful verification tool. It allows practitioners to assess the amount of tooth structure removed and make adjustments in under-prepared areas. 

On the other hand, Luo et al. and Gao et al. developed a more complex stereolithographic template with multiple depth-guiding holes 1.2 mm in diameter planned at designated points on the labial surface and incisal edge of the teeth [30,31]. This design includes a channel for a calibrated bur with depth scales and stopper, which create dimples on the labial surfaces of the tooth. The dimples are then marked with a pencil as depth guides for subsequent tooth preparation. The use of this template design has been associated with a reported depth deviation approximately 0.3mm from the planned tooth preparation depth [31]. This deviation may be attributed to inaccuracies introduced during the subsequent free-hand tooth reduction, despite the template’s precise depth-guiding features. 

On the contrary, Silva et al. employed commercially available First-fit software to virtually design a series of 6 to 8 tooth reduction templates for PLVs [6]. This software enables the creation of sequential templates that guide the tooth preparation process, starting from the incisal preparation, followed by the incisal third of the labial surface and middle third of the labial surface. Additionally, the system includes a specially designed handpiece that fits into the rotary instrument access window within the templates, allowing for precise movement in the desired direction and enhancing the accuracy of tooth structure removal. However, this system does not offer guidance for tooth reduction in interproximal areas and for defining the finish line preparation. 

On the other hand, Marques et al. developed a series of five tooth reduction templates for each stage of tooth preparation for PLV. Despite the smaller number of templates used as compared to those templates developed by Silva et al. [6], these templates provide comprehensive guidance for various aspects of tooth reduction, including the incisal edge, incisal third and the middle third of labial surface, as well as the interproximal and cervical finishing line [10]. A rotary instrument access window was incorporated in the templates for the labial surface and the interproximal and cervical finishing line preparations, which facilitates precise control over bur movement during tooth preparation procedure. However, their design does not require a specially designed handpiece, significantly reducing the equipment cost.

### 4.5. Stereolithographic Template Fabrication

Advancements of digital technology have enabled the fabrication of tooth reduction templates using additive manufacturing methods. Compared to subtractive manufacturing methods, additive methods offer faster fabrication [37,38,39] and comparable accuracy [40,41]. Various 3D printing technologies including Digital Light Processing (DLP) [10], Stereolithography (SLA) [6], Liquid Crystal Display (LCD) [28,29] and multi-jet printing (MJP) technology [30,31] have been employed in the included studies to fabricate tooth reduction stereolithographic templates with a precision range from 25.4 μm to 62 μm. 

The accuracy of stereolithographic template fabrication is significantly influenced by the type of printing technology employed. However, results across studies are inconclusive due to variations in study designs. DLP and SLA are commonly used vat polymerization technologies in 3D printing, each using different methods for resin polymerization. Le at al. demonstrated that implant surgical templates printed using DLP technologies achieved higher accuracy than those produced with SLA technology in their in vitro study [42]. LCD is a newer 3D printing technology that employs light-emitting diodes (LEDs) projected in parallel directly onto the build area, enhancing the precision of stereolithography templates. However, its precision can be compromised by high-temperature sterilization (121 °C and 134 °C). Instead, Labakoum et al. recommended using 70% isopropyl alcohol for disinfecting stereolithographic template to maintain accuracy [43]. On the other hand, MJP technology, which uses piezo printhead technology to deposit photopolymer resin layer by layer has been shown to be less accurate compared to SLA for fabricating stereolithographic templates [44]. While printing technologies are crucial, other factors such as printing orientation, the placement of supports and slice layer thickness also play a significant role in determining the accuracy of stereolithographic templates. Nevertheless, these factors are not well addressed in the studies included.

Three-dimensional printing uses photopolymerizable resins that are cured layer by layer at their predetermined thickness into the final structure. Of the articles included in this review, some did not mention the type of resin used [6,11], while four articles used non-biocompatible resins [28,29,30,31] and only one article utilized biocompatible resins [10]. Currently, there are no specific guidelines addressing the biocompatibility of resins used for temporary applications [45], such as guided tooth reduction templates. However, for long-term applications, it is important to consider biocompatible 3D printing PMMA resins, including those incorporating bioactive glass for their enhanced safety [46]. Nevertheless, for guided PLV preparation, the stereolithographic templates must be precise, stable in the mouth and rigid for optimal precision. Transparent resins are preferred due to their ability to provide adequate visualization of the prepared tooth and improved illumination. 

### 4.6. Limitations

As is inherent to the design of a scoping review, our study presents several limitations. First, this scoping review is limited by the inclusion of a relatively small number of studies addressing computer-assisted veneer preparation, all of which are case reports and case series. The included studies had a high level of heterogeneity due to different digital technologies employed; thus, a standardized protocol in virtual patient generation could not be established. This lack of standardization negatively impacts the accuracy of stereolithographic templates used in veneer preparation. Additionally, only one study compared the actual tooth preparation depth with the planned depth, highlighting a gap in verifying the accuracy for computer-assisted veneer preparation workflows using stereolithographic templates in the other studies.

### 4.7. Future Directions

The emergence of Artificial Intelligence (AI) holds significant potential to streamline the workflow of computer-assisted veneer preparation by enhancing various stages of the process. The development of a robotic systems powered by AI can assist dental clinicians to precisely execute veneer preparation according to pre-defined trajectory plans. This advancement helps address issues related to the inaccuracy of manual tooth preparation in the final stage of computer-assisted veneer preparation [47]. Moreover, incorporating augmented reality (AR) technology into robotic systems could provide clinicians with a visual overlay of the planned tooth preparation information, improving both the visualization and the efficiency of veneer preparation [48]. As this technology begins to look promising, its clinical applications and benefits will have to be strongly established by clinical trials. Nevertheless, the role of AI and AR should not be neglected in the development of technology in dentistry with the aim to improve precision, accuracy and improved tissue preservation. 

## 5. Conclusions

The digital workflows for designing and fabricating stereolithographic templates for PLV preparation are inconsistent due to variabilities in software, scanning and printing technologies, and a wide range of template designs and quantities utilized across different studies. Further randomized clinical trials are warranted to establish a standardized protocol and validate workflows for computer-assisted veneer preparation. 

## Figures and Tables

**Table 1:** Keywords in different databases for literature searching.

Database	Keywords
Web of Science	TS = (((veneers) OR (porcelain laminated veneers) OR (ceramic veneers)) AND ((printed) OR (templates) OR (manufactured) OR (guided) OR (assisted) OR (designed) OR (digital)) AND ((tooth reduction) OR (tooth preparation)))
PubMed	TS = (((veneers) OR (porcelain laminated veneers) OR (ceramic veneers)) AND ((printed) OR (templates) OR (manufactured) OR (guided) OR (assisted) OR (designed) OR (digital)) AND ((tooth reduction) OR (tooth preparation)))
Scopus	({veneers} OR {porcelain laminated veneers} OR {ceramic veneers}) AND ({printed} OR {templates} OR {manufactured} OR {designed} OR {digital} OR {guided} OR {assisted}) AND ({tooth reduction} OR {tooth preparation}) AND PUBYEAR > 2013 AND PUBYEAR < 2025 AND (LIMIT-TO (DOCTYPE, “ar”)) AND (LIMIT-TO (LANGUAGE, “English”)) AND (LIMIT-TO (EXACTKEYWORD, “Computer Aided Design”) OR LIMIT-TO (EXACTKEYWORD, “Computer-Aided Design”) OR LIMIT-TO (EXACTKEYWORD, “Dental Veneers”) OR LIMIT-TO (EXACTKEYWORD, “Dental Veneer”) OR LIMIT-TO (EXACTKEYWORD, “CAD/CAM”) OR LIMIT-TO (EXACTKEYWORD, “Three Dimensional Printing”) OR LIMIT-TO (EXACTKEYWORD, “Workflow”) OR LIMIT-TO (EXACTKEYWORD, “Computer Aided Design/computer Aided Manufacturing”) OR LIMIT-TO (EXACTKEYWORD, “3D Printing”) OR LIMIT-TO (EXACTKEYWORD, “CAD-CAM”) OR LIMIT-TO (EXACTKEYWORD, “Computer-aided Design”) OR LIMIT-TO (EXACTKEYWORD, “Cad/cams”) OR LIMIT-TO (EXACTKEYWORD, “Additive Manufacturing”) OR LIMIT-TO (EXACTKEYWORD, “Computer Aided Manufacturing”) OR LIMIT-TO (EXACTKEYWORD, “Three Dimensional Imaging”) OR LIMIT-TO (EXACTKEYWORD, “Imaging, Three-Dimensional”) OR LIMIT-TO (EXACTKEYWORD, “Printing, Three-Dimensional”) OR LIMIT-TO (EXACTKEYWORD, “Tooth Preparation”))

**Table 2:** The characteristics of the seven case reports and a summary of the digital workflow for the esthetic pre-evaluative temporary approach in computer-assisted porcelain laminate veneer preparation.

Authors and Year	Types of Study	Number of Teeth (Tooth Type)	Types of Scanners	STL Diagnostic Data Acquisition	Virtual Patient Model	Digital Wax-Up	Aesthetic Validation Procedure
Silva et al., 2020 [6]	Case report	Case 1: 10 teeth (teeth 15 to 25)	Case 1: Extraoral scanner (3Shape D2000)	Case 1: Indirect scans of maxillary and mandibular cast	Case 1: Maxillary cast scan	Case 1: Digital wax-up was made on maxillary digital cast	Case 1: Mock-up was completed using three different digital wax-ups
		Case 2: 10 teeth (teeth 15 to 25)	Case2: Intraoral scanner (3Shape Trios3)	Case2: Direct scans of Maxillary and mandibular dentitions	Case 2: Intraoral scans	Case 2: Digital wax-up was created using Digital Smile Design planning software	Case 2: Mock-up was completed using a silicone index taken from a printed wax cast and Luxatemp Bisacryl
Gao et al., 2020 [31]	Case report	8 teeth (teeth 14 to 24)	- Intraoral scanner (3Shape TRIOS) - Face scanner (3DMD) - CBCT (3D Accui tomo 170)	- Direct scans of maxillary and mandibular dentition - Facial scan - CBCT scan of maxilla skeletal	Superimposition of intraoral, facial and CBCT scans using Exocad software 2018	Digital wax-up was created on virtual patient mounted on virtual articulator	Not completed
Luo et al., 2022 [30]	Case report	16 teeth (teeth 11 to 24, teeth 34 to 44)	- Intraoral scanner (Trios Color Pod) - CBCT (3D Accui tomo 170) - Face scanner (3dMDs)	- Direct scans of maxillary and mandibular dentition - Facial scan - CBCT scan of maxilla and mandible skeletal	Superimposition of CBCT, intraoral and face scans using Exocad software 2018	Digital wax-up was created on virtual patient mounted on virtual articulator	Not completed
Tinoco et al., 2023 [28]	Case report	10 teeth (teeth 15 to 25)	Laboratory scanner (Degree of Freedom HD)	Indirect scans of maxillary and mandibular casts	Diagnostic casts scan	Not mentioned	Intraoral mock-up was performed using Structure Premium
Robles et al., 2023 [29]	Case report	8 teeth (teeth 14 to 24)	Laboratory scanner (Degree of Freedom HD)	Indirect scans of maxillary and mandibular casts	Diagnostic casts scan	Digital wax-up was created on digital diagnostic casts	Diagnostic mock-up was performed using temporary bis-acrylic material
Figueira et al., 2023 [11]	Case report	10 teeth (teeth 15 to 25)	-Intraoral scanner (3Shape) -Extraoral photog raphs (Kois Facial Reference Glasses) -CBCT	-Direct scans of maxillary and mandibular dentition -Indirect scans of diagnostic casts	Superimposition of extraoral photographs and intraoral scans	Diagnostic wax-up was created on virtual patient	Diagnostic mock-up was performed using Bonded Functional Esthetic Prototype (BFEP)
Marques et al., 2024 [10]	Case report	6 teeth (teeth 13 to 23)	Intraoral scanner (CS3600; Carestream)	Direct scans of maxillary and mandibular dentition	Superimposition of extraoral photographs and intraoral scans	Digital wax-up was created on digital diagnostic casts	Trial restoration was performed using bis-acrylic resin

**Table 3:** The digital workflow of designing and manufacturing tooth reduction stereolithographic templates along with their limitations.

Authors and Years	CAD Software Used for Template Designs	Virtual Tooth Preparation	Number of Templates	Template Designs	Types of 3D Printer	Types of Template Resins	Limitations of the Template Design
Silva et al., 2020 [6]	First Fit software	Virtual tooth reduction was performed from digital wax-up using First Fit software	Case 1: 6	Each template was designed with a window slot to engage the special First Fit handpiece	Formlabs 2	Not mentioned	1. A specially designed bur was required 2. No guidance for tooth reduction in interproximal areas and the cervical finish line
			Case 2: 8				
Gao et al., 2020 [31]	Exocad 2018	Desired volume was reduced from digital wax-up using “off-set” tool	1	Template was designed with cylindrical guide tubes on labial and incisal surfaces	ProJet MJP 3600 MultiJet	Resin (VisiJet S300)	1. Calibrated bur with stopper was required 2. Only provides depth groove for subsequent ve neer preparation
Luo et al., 2022 [30]	Exocad 2018	Not mentioned	1	The templates were designed with tubes at designated points on labial, incisal and palatal surfaces	ProJet MJP 3600 MultiJet	Resin (VisiJet S300)	1. Calibrated bur with stopper was required 2. Only provides depth groove for subsequent ve neer preparation
Tinoco et al., 2023 [28]	Exocad version 2.4	Not mentioned	1	Template incorporated a cross-shaped design	Mono 4K, Anycubic	Transparent photo-polymerizable resin (Anycubic Clear UV Resin)	Only provide depth groove for subsequence veneer preparation
Robles et al., 2023 [29]	Exocad (Exocad 2.4)	Not mentioned	1	Template incorporated a cross-shaped design	Mono 4K, Anycubic	Transparent photopolymerizable resin (Anycubic Clear UV Resin)	Only provides depth groove for subsequent veneer preparation
Figueira et al., 2023 [11]	3Shape	Virtual tooth preparation is performed using virtual burs	1	An open window was designed on the labial surface of template	Not mentioned	Not mentioned	Free-hand tooth preparation at the cervical finish line is required
Marques et al., 2024 [10]	Meshmixer	Desired volume was reduced from digital wax-up	5	Each template was designed with open access to the labial surfaces of the prepared tooth and rotary instrument access sleeved windows	Max UV. Asiga (DLP) 62 μm	Clear resin (Freeprint Ortho; Detax).	Free-hand polishing is needed after guided tooth preparation

**Table 4:** Quality assessment of the included case reports using the Joanna Briggs Institute (JBI) critical appraisal checklist for case reports.

Authors and Year	A1	A2	A3	A4	A5	A6	A7	A8	Total Yes (Max 8)
Gao et al., 2020 [31]	Yes	No	Yes	Yes	Yes	Yes	Unclear	Yes	6
Luo et al., 2022 [30]	Yes	Yes	Yes	Yes	Yes	Yes	No	Yes	8
Tinoco et al., 2023 [28]	Yes	No	Yes	Yes	Yes	Yes	No	Yes	6
Robles et al., 2023 [29]	Yes	No	Yes	Yes	Yes	Yes	No	Yes	6
Figueira et al., 2023 [11]	Yes	No	Yes	Yes	Yes	Yes	No	Yes	6
Marques et al., 2024 [10]	Unclear	No	Yes	Yes	Yes	Yes	No	Yes	5

Answer options include: “Yes”, “No”, “Unclear” or “Not applicable”; B1: Were there clear criteria for inclusion in the case series?; B2: Was the condition measured in a standard, reliable way for all participants included in the case series?; B3: Were valid methods used for identification of the condition for all participants included in the case series?; B4: Did the case series have consecutive inclusion of participants?; B5: Did the case series have complete inclusion of participants?; B6: Was there clear reporting of the demographics of the participants in the study?; B7: Was there clear reporting of clinical information of the participants?; B8: Were the outcomes or follow up results of cases clearly reported?; B9: Was there clear reporting of the presenting site(s)/clinic(s) demographic information?; B10: Was the statistical analysis appropriate?

**Table 5:** Quality assessment of the included case series using the Joanna Briggs Institute (JBI) critical appraisal checklist for case series.

Authors and Year	B1	B2	B3	B4	B5	B6	B7	B8	B9	B10	Total Yes (Max 10)
Silva et al., 2020 [6]	Unclear	Yes	Yes	Unclear	Unclear	Yes	Yes	Yes	Yes	N/A	6

Answer options include: “Yes”, “No”, “Unclear” or “Not applicable”; A1: Were patients’ demographic characteristics clearly described?; A2: Was the patient’s history clearly described and presented as a timeline?; A3: Was the current clinical condition of the patient on presentation clearly described?; A4: Were the diagnostic tests or assessment methods and the results clearly described?; A5: Was the intervention(s) or treatment procedure(s) clearly described?; A6: Was the post-intervention clinical condition clearly described?; A7: Were adverse events (harms) or unanticipated events identified and described?; A8: Does the case report provide takeaway lessons?

## Data Availability

Not applicable.
